# Intra- and Inter-examiner Reliability of Diaphragmatic Ultrasound Parameters in People with Stable Chronic Obstructive Pulmonary Disease

**DOI:** 10.1298/ptr.26-E00013

**Published:** 2026-06-25

**Authors:** Takako TANAKA, Kenneth WU, Yorihide YANAGITA, Tatsuya MORISHITA, Shusuke TOYAMA, Fumiya NAGATA, Kazuya SHINGAI, Tomoyuki IKEUCHI, Kentaro MORI, Kazuaki SUYAMA, Sunita MATHUR, Ryo KOZU

**Affiliations:** 1 Department of Physical Therapy Science, Nagasaki University Graduate School of Biomedical Sciences, Japan; 2 Department of Rehabilitation Medicine, Tagami Hospital, Japan; 3 Department of Pulmonary Rehabilitation, Masaki Pulmonary Clinic, Japan; 4 Department of Medicine, University of Toronto, Canada; 5 Division of Respirology, St. Michael's Hospital, Unity Health Toronto, Canada; 6 Pulmonary Rehabilitation Center, Kirigaoka Tsuda Hospital, Japan; 7 Department of Physical Therapy, Faculty of Rehabilitation, Reiwa Health Sciences University, Japan; 8 School of Rehabilitation Therapy, Queen’s University, Canada

**Keywords:** Chronic obstructive pulmonary disease, Diaphragm, Ultrasound, Reliability

## Abstract

**Objectives:**

This study aimed to evaluate the intra- and inter-examiner reliability of ultrasound-derived diaphragm thickness (Tdi) and diaphragmatic excursion (DE) in individuals with chronic obstructive pulmonary disease (COPD).

**Methods:**

This prospective cross-sectional study included outpatients with stable COPD. Six participants were analyzed for intra-examiner reliability and 16 for inter-examiner reliability. Two trained examiners performed diaphragmatic ultrasound using a same-day standardized protocol. In the intra-examiner phase, 1 examiner performed 2 repeated measurements in 6 participants on the same day. In the inter-examiner phase, 2 examiners performed 2 measurements in 16 participants under the same-day standardized protocol. Tdi was measured at functional residual capacity (FRC), residual volume (RV), and total lung capacity (TLC), and DE was measured from resting expiration to maximal inspiration. Intra-examiner reliability was assessed using intraclass correlation coefficients (ICCs; ICC [1,1]), and inter-examiner reliability was evaluated using ICC (2,1); standard error of measurement and minimal detectable change were also calculated.

**Results:**

Intra-examiner ICCs ranged from 0.70 to 0.98. Tdi at TLC (Tdi_TLC_) and DE demonstrated high intra-examiner reliability (ICCs ≥0.83 for both examiners). Inter-examiner analysis showed high agreement for Tdi_TLC_ and DE (ICCs = 0.83 and 0.88, respectively), whereas Tdi at FRC and RV showed moderate agreement (ICCs = 0.57 and 0.59, respectively).

**Conclusions:**

Ultrasound-derived diaphragm parameters—particularly Tdi_TLC_ and DE—demonstrated high intra- and inter-examiner reliability in individuals with stable COPD under a same-day standardized protocol. This suggests the potential clinical utility of diaphragmatic ultrasound for the reliable evaluation and short-term monitoring of diaphragmatic function in clinical and research settings.

## Introduction

Chronic obstructive pulmonary disease (COPD) is a common respiratory disorder characterized by persistent airflow limitation. Exertional dyspnea is a primary symptom of COPD and progressively impairs physical function and quality of life. Exertional dyspnea is partly caused by diaphragmatic dysfunction^1)^, as lung hyperinflation shortens the diaphragm in individuals with COPD, which reduces its mechanical efficiency. In severe COPD, systemic inflammation induces structural and molecular alterations in the diaphragm, including decreased myosin content and sarcomere damage, further impairing diaphragmatic function^2,3)^. Importantly, diaphragmatic dysfunction in COPD is not static and may be modifiable through pulmonary rehabilitation and inspiratory muscle training^4,5)^. Therefore, accurate clinical assessment of diaphragmatic function is essential to monitor its status throughout and after pulmonary rehabilitation in individuals with COPD.

Diaphragmatic ultrasound (US) is a simple, noninvasive, and real-time assessment tool^6)^. A recent review reported the rapid expansion of diaphragmatic US in both clinical and research settings, with multiple measurement approaches proposed beyond traditional single indicators^7)^. This increase underscores the need to establish the reproducibility of commonly used diaphragmatic US parameters.

Diaphragm thickness (Tdi) and diaphragmatic excursion (DE) are commonly used in diaphragmatic US assessment. While high intra- and inter-examiner reliability of these parameters has been consistently demonstrated in healthy individuals^8–12)^, the reliability of these parameters in people with COPD remains inadequately examined. Although the intra-examiner reliability of Tdi in individuals with COPD has been reported to be comparable to that in healthy populations^13,14)^, Tdi measurements are influenced by interindividual factors, including body composition and the posture of the individual at the time of diaphragmatic US assessment^15–19)^. Similarly, although the intra-examiner reliability of DE has been reported to show good reproducibility (intraclass correlation coefficient [ICC] (1,1) = 0.89; ICC (1,k) = 0.91)^20)^, the measurement is sensitive to breathing patterns, and its inter-examiner reliability in COPD has not been sufficiently investigated. To date, no study has simultaneously evaluated the reproducibility of Tdi and DE in individuals with COPD using standardized measurement conditions within a single study design. Keeping these factors constant is crucial, as these parameters reflect distinct physiological components of diaphragmatic function—Tdi representing structural characteristics and DE representing its excursion during breathing^21)^. This study aimed to assess and compare the intra- and inter-examiner reproducibility of US-derived Tdi and DE in individuals with COPD under standardized conditions.

## Methods

### Study design

This is part of a larger prospective cross-sectional study, approved by the Human Ethics Review Committee of the Nagasaki University Graduate School of Biomedical Sciences (approval number 24041104-2). The study was conducted in 2 sequential phases, with Phase 1 (intra-examiner reproducibility) followed by Phase 2 (inter-examiner reproducibility).

### Participants/eligibility criteria

Participants were recruited from the outpatient clinics of Masaki Pulmonary Clinic, Tagami Hospital (Nagasaki, Japan) and Kirigaoka Tsuda Hospital (Fukuoka, Japan) between September 2024 and February 2025. Two separate cohorts participated in the 2 phases of the study. Participants were included if they were male, at least 65 years old; had a diagnosis of COPD by a respiratory physician and a record of spirometry results confirming obstructive airway disease; were on medical management for their COPD; and were clinically stable (i.e., no acute exacerbation of their COPD within the past 6 weeks)^22)^. Only male participants were included to minimize biological variability related to sex differences in Tdi and diaphragmatic excursion^16,23)^, thereby enhancing methodological consistency in this reproducibility study. Individuals were excluded if they were unable to lie supine or stand for testing, had a comorbidity (i.e., neuromuscular diseases) that affected diaphragmatic functioning, or used a pacemaker.

### Diaphragmatic US measurements protocol

A Vscan Air CL (GE HealthCare, Chicago, IL, USA) was used for diaphragmatic US image acquisition. In Phase 1, participants underwent 4 sequential US assessments in which Tdi and DE were measured. Each assessment was separated by a 30-min rest period. Examiner 1 performed the first and third assessments, and Examiner 2 performed the second and fourth assessments. In Phase 2, participants underwent 2 sequential assessments, also separated by a 30-min rest period, with the same measurements obtained. Tdi was measured in a supine position. At least 3 images were acquired during separate breaths at the end of resting exhalation (i.e., functional residual capacity [FRC]), at maximal exhalation as much as possible (i.e., residual volume [RV]), and at maximal inhalation as much as possible (i.e., total lung capacity [TLC]). DE was measured in a standing position. This posture was chosen in accordance with previous research because it allows for evaluation under physiological conditions. Furthermore, an upright posture may reduce the impact of abdominal organ compression on diaphragmatic movement^24)^. Participants received instructions on standardized breathing techniques before the measurements. Specifically, they were instructed to perform diaphragmatic breathing, including resting breaths, maximum inhalation, and maximum exhalation. These breathing techniques were practiced a few times before data collection. During practice and measurement, the examiner visually confirmed abdominal expansion to ensure that proper diaphragmatic breathing was performed. At least 3 images were obtained while the participant performed a maneuver from resting expiration to maximum inspiration. The image acquisition timing was determined in real time by visually confirming the phasic diaphragm movement on the US monitor in accordance with the participant’s breathing pattern. To minimize landmarking discrepancies, the first examiner marked the probe position, which was reused for all subsequent US acquisitions. The acquisition settings (frequency, depth, and gain) were also kept constant for each participant. All images were analyzed using the Vscan Air CL built-in software (GE Healthcare Japan).

### Tdi and %∆Tdi assessment

Tdi was assessed with B-mode setting, fitted with a 3–12-MHz linear probe. The US settings were standardized at depths of 3.5–6 cm and a gain of 60 dB. The participants were positioned supine with their legs bent and supported by a cushion. The probe was placed perpendicular to the diaphragmatic attachment (zone of apposition) on the chest wall in the 8th or 9th right intercostal space between the anterior and mid-axillary lines. The diaphragm was visualized as an intermediate space between the pleural and peritoneal lines at the end of expiration^25)^. Tdi was measured to the nearest 0.1 mm using a still-image electronic caliper as the distance from the deep peritoneal border to the superficial diaphragmatic pleural border (Fig. 1A).

**Fig. 1.**
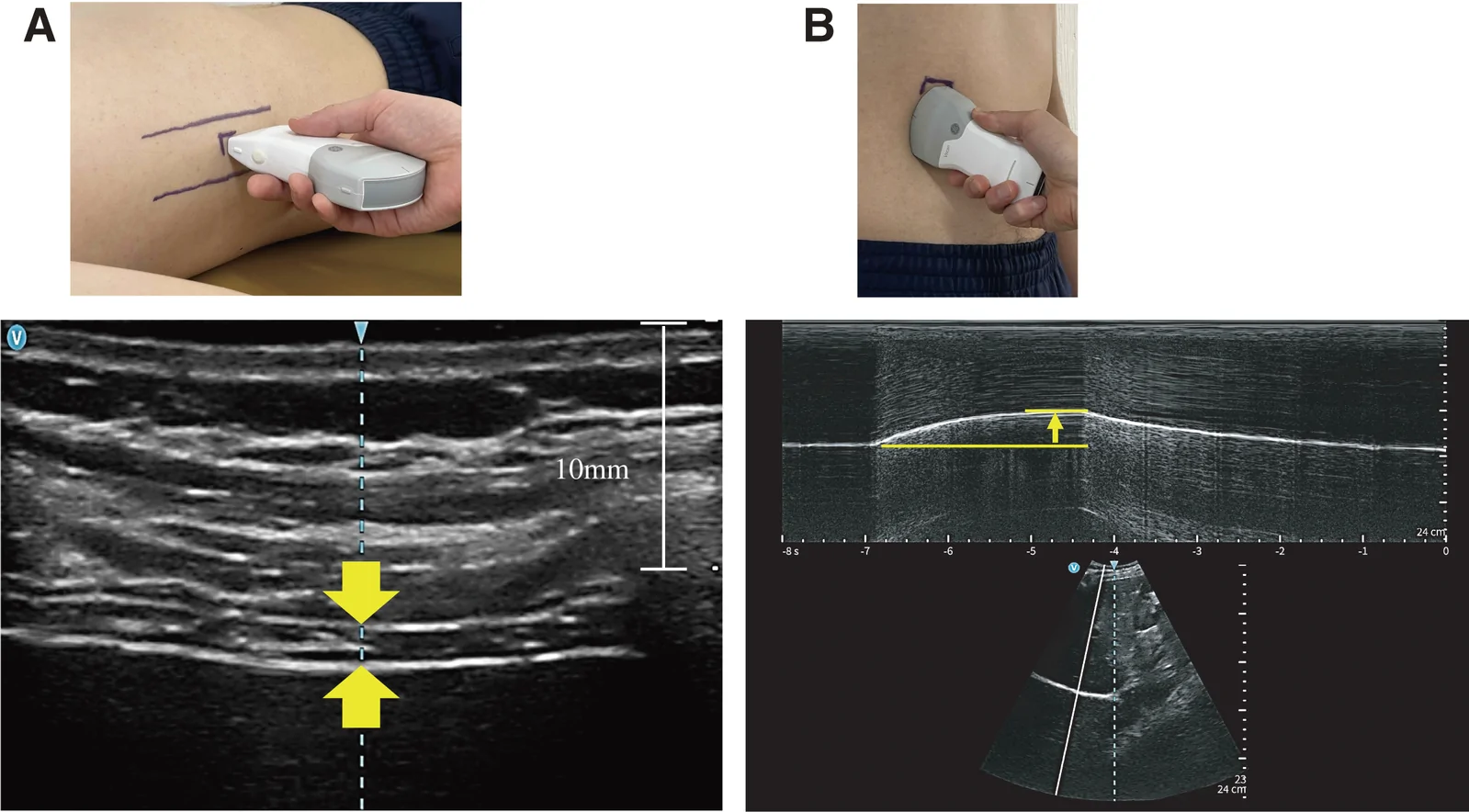
Ultrasound image demonstrating measurements of (**A**) diaphragmatic muscle thickness and (**B**) diaphragmatic excursion. Representative ultrasound images demonstrating measurements of (**A**) Tdi and (**B**) DE. Tdi was measured in B-mode imaging, and DE was assessed using M-mode from resting expiration to maximal inspiration. Arrows in panel **A** indicate the pleural and peritoneal membranes used for measurement of diaphragmatic muscle thickness. The arrow in panel **B** indicates maximum DE during deep breathing (DEmax). Tdi, diaphragm thickness; DE, diaphragmatic excursion; DEmax, maximum diaphragmatic excursion

### DE assessment

DE was recorded in M-mode using a 2–5-MHz convex probe. The US settings were standardized at a depth of 8 cm and a gain of 60 dB. The participants were positioned standing with their backs against the wall and their muscles relaxed for the US. The probe was placed longitudinally along the anterior axillary line, below the right costal arch. The M-mode beam was aligned at an angle of approximately 30° (range: 0°–45°) relative to the craniocaudal midline of the trunk and positioned over the right hemidiaphragm. DE was assessed as the displacement from RV to maximal inspiration to the nearest 0.1 mm using a still-image electronic caliper (Fig. 1B)^26)^.

### Examiner training

Two examiners (both physiotherapists) were trained by an expert physiotherapist and a physician with over 15 years of experience in diaphragmatic US acquisition and analysis. Both trainers had extensive clinical and research experience in diaphragmatic US. The examiners were physical therapists with clinical experience in pulmonary rehabilitation who had no prior experience with diaphragmatic US examinations but were interested in acquiring this technique. Two physical therapists with extensive clinical experience (25 and 17 years) were selected as candidates. Both examiners had approximately equivalent years of clinical and practical experience in pulmonary rehabilitation. The examiners practiced the technique with 15 healthy adult male volunteers over 50 practice sessions.

### Demographic and clinical data

Demographic variables and spirometry data were collected from the participant’s clinic charts. Spirometry variables were extracted from the most recent spirometry performed within 6 months prior to the US assessment as part of routine clinical care, including forced expiratory volume in 1 second (FEV_1_), forced vital capacity (FVC), and the FEV_1_/FVC ratio (absolute value and/or percentage predicted). Disease severity was defined based on the percentage predicted FEV_1_, and it was categorized according to the Global Initiative for Chronic Obstructive Lung Disease (GOLD) criteria^27)^. Modified Medical Research Council dyspnea scale scores were also recorded at the most recent outpatient visit within the same 6-month period and were also extracted from the medical chart.

### Statistical analysis

The sample size was calculated separately for the 2 phases of this study. For Phase 1 (intra-examiner reproducibility), the sample size calculation was based on ICC values reported by Okura et al.^13)^ and Shiraishi et al.^20)^ using ICC with α = 0.05, 1 – β = 0.80, and k = 2. The number of participants required for this phase was n = 6. For Phase 2 (inter-examiner reproducibility), the sample size was determined based on a clinically acceptable reliability threshold. Specifically, the null hypothesis was defined as ICC <0.80, whereas the expected ICC was set at 0.95, consistent with previously reported inter-examiner reliability for diaphragmatic US^28)^. Under these assumptions (α = 0.05, 1 – β = 0.80, k = 2), the required sample size was estimated to be n = 16.

All data are expressed as the mean ± standard deviation (SD) for continuous variables and as frequency for categorical variables. Intra-examiner reproducibility was assessed using ICC (1,1), and inter-examiner reproducibility was evaluated using ICC (2,1). ICC values of 0.81–1.0 represented almost perfect or perfect agreement, 0.61–0.80 substantial agreement, 0.41–0.60 moderate agreement, 0.21–0.40 fair agreement, and 0.20 or below slight agreement^29)^. The standard error of measurement (SEM) was calculated using the following formula: SEM = SD_pooled_ × √(1 − ICC), where SD_pooled_ is the pooled SD of the test and retest measurements. The minimal detectable change at the 95% confidence level (MDC_95_) was computed using the following formula: MDC_95_ = 1.96 × SEM × √2 to determine the smallest change that can be interpreted as a real change beyond measurement error. All calculations and statistical analyses were performed using IBM SPSS Statistics version 27.0 for Windows (IBM, Chicago, IL, USA) and Microsoft Excel 365 (Microsoft, Redmond, WA, USA). Statistical significance was defined as *p* <0.05.

## Results

### Participant characteristics

Six participants were recruited and performed in Phase 1 of this study, and 16 were recruited and participated in Phase 2. The age (mean ± SD) was 77.8 ± 6.4 in Phase 1 and 76.9 ± 5.5 in Phase 2. Demographic and clinical characteristics are summarized in Table 1.

**Table 1.** Demographic and clinical characteristics

Characteristics	Mean ± SD
Phase 1	
Age (year)	77.8 ± 6.4
Spirometry	
FVC (% predicted)	81 ± 23
FEV_1_ (% predicted)	57 ± 23
FEV_1_/FVC (%)	54 ± 12
GOLD stage: I/II/III/IV (frequency)	1/3/2/0
mMRC dyspnea scale: 0/1/2/3/4 (frequency)	0/2/2/2/0
Phase 2	
Age	76.9 ± 5.5
Spirometry	
FVC (% predicted)	82 ± 26
FEV_1_ (% predicted)	54 ± 21
FEV_1_/FVC (%)	66 ± 13
GOLD stage: I/II/III/IV (frequency)	2/6/5/3
mMRC dyspnea scale: 0/1/2/3/4 (frequency)	0/4/6/5/1

SD, standard deviation; FVC, forced vital capacity; FEV_1_, forced expiratory volume in 1 second; GOLD, Global Initiative for Chronic Obstructive Lung Disease; mMRC, modified Medical Research Council

### Phase 1: Intra-examiner reproducibility

The intra-examiner reliability of diaphragmatic US (ICC from both examiners) ranged from 0.70 to 0.98, with the ICC of Tdi obtained at TLC (Tdi_TLC_) and DE above 0.81 in both the examiners. However, the lower bound of the 95% confidence interval (CI) for Tdi_TLC_ in Examiner 2 indicated only moderate reliability (Table 2). In contrast, DE showed consistently narrow 95% CIs in both examiners.

**Table 2.** Means, standard deviations, and intra-examiner reliability of diaphragmatic ultrasound (n = 6)

Examiners	Test	Retest	ICC (1,1)	95% CI	MDC_95_
Examiner 1					
Tdi_FRC_ (mm)	1.63 ± 0.25	1.62 ± 0.20	0.84^***^	0.56–0.95	0.247
Tdi_RV_ (mm)	1.53 ± 0.22	1.52 ± 0.15	0.81^***^	0.47–0.94	0.227
Tdi_TLC_ (mm)	3.26 ± 0.46	3.33 ± 0.51	0.94^***^	0.82–0.98	0.319
DE (cm)	4.20 ± 1.28	4.42 ± 1.18	0.98^***^	0.94–1.00	0.457
Examiner 2					
Tdi_FRC_ (mm)	1.53 ± 0.22	1.57 ± 0.21	0.70^**^	0.26–0.90	0.327
Tdi_RV_ (mm)	1.45 ± 0.22	1.48 ± 0.18	0.70^**^	0.27–0.90	0.302
Tdi_TLC_ (mm)	3.14 ± 0.44	3.23 ± 0.51	0.83^***^	0.53–0.95	0.540
DE (cm)	4.33 ± 1.41	4.63 ± 1.27	0.97^***^	0.91–0.99	0.598

Data are presented as the mean ± standard deviation (SD).

^**^Substantial agreement.

^***^Almost perfect agreement.

ICC, intraclass correlation coefficient; CI, confidence interval; MDC_95_, minimum detectable change with a confidence level of 95%; Tdi_FRC_, diaphragm thickness at functional residual capacity; Tdi_RV_, diaphragm thickness at residual volume; Tdi_TLC_, diaphragm thickness at total lung capacity; DE, diaphragm excursion

### Phase 2: Inter-examiner reproducibility

The level of agreement between the 2 examiners differed depending on the diaphragmatic US parameter and lung volume condition (Table 3). Tdi_TLC_ and DE demonstrated high agreement between examiners (ICCs of Tdi_TLC_ and DE = 0.83 and 0.90, respectively); however, the lower bound of the 95% CI for Tdi_TLC_ indicated only moderate reliability. Measurements obtained at FRC and RV showed only moderate inter-examiner reliability (ICCs of Tdi_FRC_ and Tdi_RV_ = 0.57 and 0.59, respectively).

**Table 3.** Means, standard deviations, and inter-examiner reliability of diaphragmatic ultrasound (n = 16)

	Examiner 1	Examiner 2	ICC (2,1)	95% CI	MDC_95_
Tdi_FRC_ (mm)	1.66 ± 0.24	1.57 ± 0.20	0.57^*^	0.17–0.82	0.205
Tdi_RV_ (mm)	1.54 ± 0.21	1.47 ± 0.20	0.59^*^	0.19–0.83	0.363
Tdi_TLC_ (mm)	3.12 ± 0.49	3.01 ± 0.45	0.83^***^	0.59–0.93	0.540
DE (cm)	4.38 ± 1.32	4.51 ± 1.64	0.90^***^	0.70–0.95	1.354

Data are presented as the mean ± standard deviation (SD).

^*^Moderate agreement.

^***^Almost perfect agreement.

ICC, intraclass correlation coefficients; CI, confidence interval; MDC_95_, minimum detectable change with a confidence level of 95%; Tdi_FRC_, thickness of diaphragm at functional residual capacity; Tdi_RV_, thickness of diaphragm at residual volume; Tdi_TLC_, diaphragm thickness at total lung capacity; DE, diaphragm excursion

## Discussion

This is the first study to evaluate the intra- and inter-examiner reproducibility of diaphragmatic US-derived parameters under same-day standardized conditions in men with stable COPD. We found that Tdi_TLC_ and DE demonstrated consistently high reproducibility across both intra- and inter-examiner assessments, whereas measurements obtained at lower lung volumes showed greater variability. The same-day evaluation minimized physiological fluctuations and strengthened the methodological rigor of the reproducibility assessment. Collectively, these results support the clinical utility of diaphragmatic US, particularly with Tdi_TLC_ and DE, for reliable evaluation and short-term monitoring of diaphragmatic function, such as detecting within-day changes or responses to a single rehabilitation session in individuals with stable COPD.

In Phase 1, intra-examiner test–retest analyses demonstrated perfect agreement for all parameters, with the exception of Tdi_RV_ and Tdi_FRC_ in Examiner 2, which exhibited substantial agreement, indicating overall acceptable intra-examiner reproducibility. Compared with a previous study in Japanese individuals with COPD with similar measures^13)^, the current study demonstrated lower Tdi values and ICCs, particularly at FRC and RV. This difference may be partly due to the older age and lower FEV_1_/FVC and %FEV_1_ predicted in our participants in this study. In more severe COPD, lung hyperinflation and altered respiratory mechanics place the diaphragm at a mechanical disadvantage, which may contribute to reduced Tdi and greater variability in measurements across lung volumes^30)^. Measurements at higher lung volumes, such as TLC, likely reflect a more consistent contractile state and excursion pattern, whereas measurements at lower lung volumes (FRC and RV) may be more susceptible to variability in end-expiratory lung volume and breathing pattern, particularly in COPD^31,32)^. However, the precise mechanisms underlying this discrepancy cannot be fully determined from the present data. The intra-examiner reproducibility of DE was comparable to that reported in a previous study of Japanese individuals with COPD^20)^, suggesting that the high reproducibility of DE reflects intrinsic methodological and physiological features of DE measurements. Importantly, interpretation based on the 95% CI provided additional insight beyond the ICC point estimates. Among all parameters, DE demonstrated the narrowest 95% CI in both examiners, followed by Tdi_TLC_, indicating higher precision and stability of these measurements under repeated testing. In contrast, Tdi_FRC_ and Tdi_RV_ showed substantially wider CIs, suggesting greater uncertainty and variability at lower lung volumes. These findings reinforce that DE and Tdi_TLC_ represent the most robust US-derived indices for intra-examiner assessment in COPD.

Inter-examiner analysis demonstrated almost perfect or perfect agreement for Tdi_TLC_ and DE, whereas Tdi_RV_ and Tdi_FRC_ showed only moderate agreement. Even during quiet breathing, diaphragm motion exhibits physiological variability in healthy individuals^31)^. At end-expiration, when the diaphragm is thinner and in a flattened, mechanically disadvantaged position^32)^, the measurement may be more susceptible to variations in respiratory effort, body motion, timing within the respiratory cycle, and differences in the instructed expiratory level^33)^. In addition, positional and methodological factors inherent to Tdi assessment may further influence measurement variability^34)^. In COPD, lung hyperinflation and air trapping may further hinder stable lung volume control and diaphragm visualization, thereby reducing image clarity and measurement accuracy^30,33–35)^. Although both examiners underwent structured training prior to data collection, their limited prior experience with diaphragmatic US may partly explain the higher MDC_95_ values observed for all Tdi parameters compared to previous studies^13)^. These findings indicate that measurements obtained at lower lung volumes remain more vulnerable to inter-examiner variability. Consideration of the 95% CI further strengthened this interpretation. DE exhibited the narrowest CI among all parameters, followed by Tdi_TLC_, whereas Tdi_FRC_ and Tdi_RV_ showed considerably wider CIs, reflecting greater inter-examiner variability and reduced measurement precision at lower lung volumes. The consistently narrow CI for DE suggests that diaphragm excursion is less susceptible to examiner-dependent technical factors and respiratory variability, supporting its role as the most reliable parameter for inter-examiner comparison and short-term clinical monitoring.

A major strength of this study is the evaluation of both intra- and inter-examiner reliability within the same COPD cohort under identical, standardized conditions. This design mirrors routine clinical practice, in which diaphragmatic US may be performed by the same or different examiners during the same-day assessment, and supports the clinical relevance of Tdi_TLC_ and DE as reliable assessment parameters. Several limitations should also be acknowledged. First, participants were recruited from only 3 clinics, and although the sample sizes in Phases 1 and 2 were sufficient to detect the ICCs, they remained relatively small, which may limit the generalizability of the findings. Future studies with larger and more diverse samples are warranted. Second, to minimize inter-examiner variation, the probe position was marked by the first examiner. Although this approach reduced landmarking discrepancies, it may have an overestimated inter-examiner reliability compared with routine clinical practice, in which landmarking and probe positioning are performed independently. Third, only male participants were included in this study. Therefore, the applicability of these findings to female individuals with COPD is not known, and further studies including female participants are needed. Finally, the present findings are limited to patients with stable COPD, and the applicability of these results to patients during acute exacerbation or other respiratory conditions remains unclear.

## Conclusions

The present study demonstrated high intra- and inter-examiner reproducibility of key diaphragmatic US parameters, particularly Tdi_TLC_ and DE, in people with stable COPD under a same-day standardized measurement protocol. These findings support the reliability and clinical applicability of diaphragmatic US for reliable evaluation and short-term monitoring in both clinical and research settings. However, the associations between diaphragmatic US parameters and clinically meaningful outcomes were not examined in this study and should therefore be interpreted with caution. Future studies involving larger, more homogeneous cohorts and outcome-based validation are warranted to further establish the clinical relevance of diaphragm US in stable COPD.
